# Hyaluronic Acid: Redefining Its Role

**DOI:** 10.3390/cells9071743

**Published:** 2020-07-21

**Authors:** G. Abatangelo, V. Vindigni, G. Avruscio, L. Pandis, P. Brun

**Affiliations:** 1 Faculty of Medicine, University of Padova, 35121 Padova, Italy; 2 Clinic of Plastic and Reconstructive Surgery, University of Padova, 35128 Padova, Italy; vincenzo.vindigni@unipd.it (V.V.); laura.pandis@gmail.com (L.P.); 3 Department of Cardiac, Thoracic and Vascular Sciences, Angiology Unit, University of Padova, 35128 Padova, Italy; giampiero.avruscio@sanita.padova.it; 4 Department of Molecular Medicine, Histology unit, University of Padova, 35121 Padova, Italy; paola.brun@unipd.it

**Keywords:** hyaluronic acid (HA), HA receptors, osteoarthritis, tissue-engineering

## Abstract

The discovery of several unexpected complex biological roles of hyaluronic acid (HA) has promoted new research impetus for biologists and, the clinical interest in several fields of medicine, such as ophthalmology, articular pathologies, cutaneous repair, skin remodeling, vascular prosthesis, adipose tissue engineering, nerve reconstruction and cancer therapy. In addition, the great potential of HA in medicine has stimulated the interest of pharmaceutical companies which, by means of new technologies can produce HA and several new derivatives in order to increase both the residence time in a variety of human tissues and the anti-inflammatory properties. Minor chemical modifications of the molecule, such as the esterification with benzyl alcohol (Hyaff-11^®^ biomaterials), have made possible the production of water-insoluble polymers that have been manufactured in various forms: membranes, gauzes, nonwoven meshes, gels, tubes. All these biomaterials are used as wound-covering, anti-adhesive devices and as scaffolds for tissue engineering, such as epidermis, dermis, micro-vascularized skin, cartilage and bone. In this review, the essential biological functions of HA and the applications of its derivatives for pharmaceutical and tissue regeneration purposes are reviewed.

## 1. Introduction

Hyaluronic acid (HA) is a long, unbranched polysaccharide composed of repeating disaccharides of D-glucuronic and *N*-acetyl-D-glucosamine with molecular weight (MW) reaching up to 2 × 10^7^ Da. It was first isolated by Karl Meyer and John Palmer in 1934 [1] from the vitreous body of the bovine eye, but its structure was described only 20 years later (1970) by Laurent [2]. In 1986 Balazs [3] proposed “hyaluronan” as an alternative to “hyaluronic acid” since, at physiological pH, the carboxyl groups of the molecule are dissociated and thus attract cations, such as Na^+^. The molecule is present in many strains of bacteria and is ubiquitous in all Vertebrates, where it is particularly abundant in the embryonic tissues and in the extracellular matrix (ECM) of adult soft connective tissues. However, it reaches its greater concentrations in the vitreous body of the eye and in the umbilical cord (Wharton’s jelly) [4]. Because of the molecule carboxyl groups, HA is negatively charged, highly hydrophilic and, at high molecular weights forms a viscous network. For its physico-chemical properties HA hydrates ECM and regulates tissue homeostasis and resistance to forces of compression. Many proteoglycans such as aggrecan interact with HA giving rise to molecule composites that occupy a huge volume and are responsible for the gel state of the matrix and for the stabilization of ECM structure. Moreover, HA forms a pericellular coat around most of the cells where it functions as a signaling molecule interacting with its binding proteins and regulating cell adhesion, migration and proliferation. High MW HA is also an essential component of synovial fluid, where it plays a fundamental role as a lubricant for the joints [4]. Therefore, HA has a key role in many physiological and pathological conditions.

Although it is considered mainly an extracellular molecule, it has also been found inside cells, in the perinuclear area of aortic smooth muscle cells during premitotic and mitotic stages and in cytoplasm structures, in relation to extracellular HA [5,6]. Its intracellular functions have not yet been fully characterized but a role in controlling cell proliferation and inflammation has been hypothesized.

The viscoelastic properties, physiological activity and biocompatibility of HA make it an ideal material for pharmacological applications, particularly in ophthalmology, rheumatology, and dermatology. Furthermore, chemical modification of the molecules yields biocompatible and biodegradable HA-based biomaterials widely used for wound covering and for tissue engineering.

## 2. HA Synthesis and Degradation

Unlike other glycosaminoglycans which are synthesized within the cell in the Golgi apparatus and then secreted externally by exocytosis, HA is synthesized by three transmembrane enzymes - HA synthetase 1 (HAS1), HA synthetase 2 (HAS2), HA synthetase 3 (HAS3) [7,8] on the inner side of the plasma membrane. The HA is then translocated to the extracellular space along with the elongation of the polymeric chain, through a pore in the HAS structures. The three HASs share 50–70% of their sequence which is highly conserved and is encoded by different genes located on different chromosomes. The three enzymes are differently-expressed during morphogenesis and pathological conditions, and generate HA with different MW [9,10]. It has been proposed that HAS3 produces molecules with lower MW (1 × 10^5^ to 1 × 10^6^ Da) than HAS1 and HAS2 that are able to generate large-sized HA (greater than 2 × 10^6^ Da). Moreover, HAS1 exhibits a slower activity than HAS2 and HAS3 [11,12]. Each of the HAS can play a role in cancer progression as discussed in the “Cancer Therapy” chapter.

The turnover of the molecule is a rapid process and its half-life varies from 12–24 h in the skin to a few minutes in the bloodstream [13]. The HA present in the blood is catabolized in lymph nodes and in the liver, while that present in the tissues is degraded outside the cell by hyaluronidase, reactive oxygen species (ROS), superoxide, nitric oxide and peroxynitrite generated in during inflamed or injured tissues, and inside the cell by the lysosomes [14]. In humans, six different hyaluronidases (Hyal) have been identified: Hyal-1, Hyal-2, Hyal-3, Hyal-4, PH-20 and Hyalp-1 [15]. Hyal-1 and Hyal-2 are the most characterized hyaluronidase, widely expressed in somatic tissues. They act in concert to degrade HA chains. On the cell surface, Hyal-2 binds and internalizes HA in vesicles where the molecules are cleaved into 20 kDa fragments. Subsequently, the fragments are further digested by Hyal-1 into tetrasaccharides [16]. PH-20 is present in sperm and is the most active mammalian hyaluronidase. Much is still unknown about the activity of Hyal-3, Hyal-4 and Hyalp1.

The equilibrium between HA synthesis and degradation has a key role in determining, not only the amount of the molecule, but also the MW of HA and, consequently, its properties. In fact, it was proposed that the HA of different molecular sizes can display different and sometimes opposing biological actions [17]. For instance, high MW HA has anti-inflammatory actions controlling the recruitment of inflammatory cells whereas the low MW molecules are pro-inflammatory and promote angiogenesis and tissue remodeling in the wound healing process [18,19]. Moreover, specific sizes of HA polymer have different effects on wound repair and recently, some authors demonstrated that a partially N-butyrylated HA derivative, BHA exerts anti-inflammatory effects and promotes wound healing [20,21].

HA has a fundamental role in regulating cell movement, both during embryonic development and in tumor progression. High MW-HA production, in the absence of fragmentation, is linked to cancer resistance, whereas low MW to tumor progression. In fact, low MW molecules enhance cell motility whereas high MW molecules inhibit cell movement [22]. The mechanism of action of different MW HA in tumorigenesis and in other pathological conditions remains largely unknown. However, it has been reported that low MW HAs can alter the clustering of cell surface HA receptors and consequently the activation of intra-cellular signaling pathways [23], as discussed in the next paragraph.

## 3. HA Cell Surface Receptors

HA is able to bind to specific proteins called hyaladherins [24,25]. Some of them are membrane proteins, such as CD44 (Cluster of Differentiation 44), RHAMM or CD168 (Receptor for Hyaluronan Mediated Motility) and LYVE1 (Lymphatic-Vessel Edothelial hyaluronan receptor 1). Additional HA receptors are matrix components such as aggrecan and other proteoglycans. The numbers and the types of members of the hyaladherins family are growing continuously. The functions of many of them have been widely described [26], while for others, their roles remain to be clarified.

After the interaction with cellular surface hyaladherins, HA activates intracellular signaling systems involved in proliferation, in differentiation, and in cell motility, as well as in degradation of the HA itself [27,28]. Among the molecules of membranes capable of binding to the HA, the CD44 and the RHAMM are considered the principal surface receptors. These molecules are not homologous proteins and can trigger different cell responses other than to the part of the cell on which they are located [29].

CD44 is the widely studied cell surface HA receptor, expressed in almost all human cell types. It is a multi-domain integral membrane glycoprotein resulting from alternative splicing of the transcript of a single highly conserved gene that gives rise to various isoforms, which in turn, may vary in function and properties [30,31]. The action of HA as a signal molecule is dependent on the affinity of CD44 for HA. This affinity, in turn, depends both on concentration and the MW of the molecule, by glycosylation of extracellular domains and/or by phosphorylation of the residues of serine [32]. In addition, CD44 is clustered by high MW HA polymers and can interact with other ligands, such as ECM molecules, growth factors, matrix metalloproteinases (MMPs) and cytokines [12]. The other surface receptor for HA is RHAMM, also known as CD168. It is present in several cell types and mediates cell migration via interactions with skeletal proteins, especially in tissue repair and inflammation processes [33]. It is expressed in several isoforms that arise from the alternative splicing of the transcript of a single gene. The surface HA-RHAMM complex plays a key role in activating signaling pathways that involve Src and other kinase protein complexes of focal adhesions [34,35]. This receptor was also found intracellularly, associated with mitochondria, microtubules and nuclei, as well as extracellularly, where it associates with CD-44 receptor [36,37]. Both types of receptors have been found in the tissues of the embryo, where the movement of cells on substrates rich in hyaluronic acid is critical. However, the deletion of both CD44 and RHAMM are not lethal for the embryo itself [38]. This implies that other matrix proteins may be involved in this process and assist the activity of CD44 and RHAMM. Lymphatic vessel endothelial hyaluronan receptor 1 (LYVE1) is a transmembrane glycoprotein that shares some similarities with CD44 but is expressed by lymphatic endothelia [39]. For this reason, it is used as a marker for distinguishing between blood and lymphatic vessels. LYVE1 is involved in the transport of HA from tissues to lymph via lymphatic endothelial cells and, indirectly, mediated leucocyte entry to lymphatic vessels [40].

## 4. Medical Applications of HA and its Derivatives.

### 4.1. HA in Osteoarthritis and in Cartilage Regeneration

Osteoarthritis (OA) is a pathology that involves the entire joint compartment and is very widespread among the population of middle and senile age. Conventional OA therapy uses pain relievers and non-steroidal anti-inflammatory drugs (NSAIDs, corticosteroids) that are effective but cause significant adverse effects and toxicities. For these reasons, visco-supplementation with HA and HA-derived biomaterials was a significant advance in the therapy of OA when it was proposed for the first time by Endre A. Balazs in 1971 [41] for the treatment of arthritis in horses and humans. In fact, HA is an intrinsic component of both articular cartilage matrix and synovial fluid with unique rheological properties that are lost in OA when the MW of HA decreases significantly (from 2–3 × 10^6^ to 6 × 10^5^) resulting in decreased fluid viscosity and cartilage disruption. In the 1980s, many HA derivatives were proposed for intra-articular injections to restore the homeostasis of the diseased synovial fluid and to protect articular cartilage from mechanical damage [42,43]. Synvisc (Hyalan G-F20) and Hyalgan [44,45], in particular, were the most widely used HA-based preparations in clinical trials. They were both safe and effective but multiple injections are usually needed. Furthermore, the effect of Hyalgan and Synvisc appears to last longer, suggesting that the molecule may interact with membrane receptors. Other studies demonstrated that Hyalgan was able to enhance human chondrocyte survival/proliferation after exposure to ROS, and this effect was mediated by the CD44/HA pathway [46,47]. Additional, commercially available HA derivatives such as Supartz_R (Seikagaku, Japan), Orthovisc and Monovisc (Anika, USA) and Durolane (Smith and Nephew, USA) contain HA with different MWs [48,49,50,51]. To prolong the effect of HA in the articular joint and, therefore, to reduce the repeated intra-articular injections, a hexadecylamide derivative (HYADD) with longer residence time, was also proposed [52].

Recently, new substances capable of exerting an anti-inflammatory effect and to improve the therapeutic activity of HA have been proposed, such as the mixture of HA and lactose-modified chitosan (Chitlac^®^). The results of in vivo and in vitro studies have demonstrated a significantly increased cartilage regeneration after the injection of this new compound in joints of animals in which OA was experimentally induced [53,54]. Furthermore, the addition of HA-Chitlac^®^ mixture to human chondrocyte cultures supplemented with triamcinolone acetonide-hydroxypropyl-β-cyclodextrin (TA-CD) preparations significantly attenuated the drug cytotoxicity, while preserving their anti-inflammatory effects, thus confirming the chondroprotective role of the HA-Chitlac^®^ mixture [55]. Some of the different HA derivatives commercially available for intra-articular injections in OA are summarized in Table 1.

Full-thickness cartilage defects that may be generated in the last stages of OA progression or after mechanical injury, may be healed only with autologous chondrocyte implantation, a cell therapy technique that is limited by the complexity of the surgical procedure. For this purpose, a three-dimensional biodegradable and biocompatible HA-based scaffold polymer (non-woven meshes) derived from the total esterification of the molecule with benzyl alcohol (Hyaff-11^®^, Fidia, Italy; Table 1), have been successfully utilized in the last few decades for culturing human chondrocytes. The resulting in vitro reconstructed cartilage was implanted in knee joints of OA patients [56]. Several studies have demonstrated that, after chondrocyte implantation, the regenerated tissue undergoes a process of maturation that leads to a hyaline tissue and not fibrous cartilage [57]. New preparations of HA for OA treatment and cartilage regeneration are continuously being proposed. However, to improve the outcome of cartilage repair, future investigations will be needed to better identify the factors that contribute to cartilage regeneration.

### 4.2. Ophthalmology

HA is an important component of the eye vitreous body, tear film, corneal epithelium and conjunctiva [58]. Thus, the molecule was first proposed by Balazs in 1980 [59] as a visco-surgical tool that replaces vitreous fluid lost during surgical ophthalmic procedures, protects from mechanical trauma and creates and maintains space for surgical manipulation. By 1982, an HA derivative, Healon^®^, was commercially available for ophthalmic applications and has become increasingly used as a therapeutic tool in the majority of surgical ophthalmic operations [60]. Up to now, in addition to Healon, other HA preparations are available in ophthalmology procedures [61].

Thanks to its viscoelastic properties HA aqueous solutions are extensively used as lubricant eye drops and to protect the surface of the cornea from dryness in the management of dry eye syndrome, a disease that results in visual disturbance and tear-film instability [62]. Up to now, a wide range of commercial tear supplements such as Systane^®^ (Alcon, USA) and Optive Fusion™ (Allergan, USA) have been developed (Table 1). Their safety and effectiveness in the management of dry eye disease were confirmed by many in vivo and in vitro studies [63,64,65,66]. By providing enhanced eye lubrication, HA also shows the potential to improve the hydration property of the contact lens and a low MW HA derivative solution has recently been proposed to improve their hydrophilicity. Indeed, it was found that it is able to prolong its wettability over time more effectively than other agents in a comparative study [67].

Another application of HA is its use in the delivery of topical ophthalmic drugs such as antibiotics and anti-inflammatory agents, to prolong their ocular residence and consequently their active effect [68,69,70]. In fact, topical agents into the eye are rapidly drained and HA combined with drugs might modulate the dose and the time of delivery. This additional application of HA derivatives opens up further opportunities for the study of medical and pharmaceutical applications of HA in ophthalmology.

### 4.3. Skin

HA is widely present in the ECM of the skin, the largest organ of the human body, and its presence is fundamental for the rheological, hygroscopic and viscoelastic properties of the tissue [71]. Although it has been shown that this polysaccharide is also associated with repair, details of the mechanisms through which it affects the repair process have not been elucidated [72]. HA has been demonstrated to play a crucial role by influencing inflammatory, proliferative, or re-modeling phases of skin healing process. It has either anti-inflammatory or pro-inflammatory properties, in relation to its molecular weight [73]. Specifically, HA binds CD44 keratinocyte receptors, leading to their differentiation and increasing their motility [74,75]. For these and many other well-known properties (excellent biocompatibility, biodegradability, durability and absence of toxicity) [76,77] researchers in recent years have focused their interest on the application of some HA-based products, such as Hyaff-11^®^, in skin tissue engineering and regenerative medicine. In particular, Hyaff-11^®^ non-woven meshes were seeded with dermal fibroblasts and endothelial cells to obtain in vitro reconstruction of endothelialized skin substitutes provided with a microcapillary network [78,79]. The endothelialized skin substitute accelerates the revascularization process at the transplant site by inoculation of the capillary-like structures with the local wound vessels [78]. In the last five years’ studies were focused on two types of HA-based compounds: hydrogels and nanofibrous scaffolds. HA-based hydrogels synthesized with chitosan [80,81], corn-starch and propolis [82] showed good properties in the wound-healing process, enhancing the proliferation of endothelial cells. Specifically, an HA and Fe^+^ complex assembled hydrogel was able to inhibit microbial infections [83]. HA and sodium alginate hydrogel were favorable to keratinocytes anchoring [84], while methacrylhydrazide-HA gel and HA-collagen-sericin gel were tested with human fibroblasts showing good cell viability [85,86]. A polycaprolactone-HA-epidermal growth factor nanofibrous scaffold with high bioactivity was proposed for wound healing applications [87]. Additionally, an HA and chondroitin sulfate nanofibrous scaffold was tested satisfactorily with human keratinocytes and fibroblasts, and therefore proposed for skin tissue engineering applications [88].

The latest studies include the use of high regenerative tools in skin tissue engineering, such as ADSC (adipose-derived stem cells) [89], solubilized amnion membrane [90] and autologous plasma-derived clot hydrogel [91]. A dermal substitute made of methacrylated gelatin and methacrylated hyaluronic acid-containing adipose-derived stem cells was tested in vivo and showed proliferative and angiogenic properties able to improve survival of tissue-engineered skin [89]. A wound-dressing device made with gelatin, chitosan and hyaluronic acid was enriched with an autologous clot hydrogel carrying mesenchymal stem-cells. This product was tested in vitro, in vivo and in a single human case. This hybrid biomaterial demonstrates high cell-viability, high biocompatibility, early regeneration capacity at four weeks and absence of signs of rejection [91]. The solubilized amnion membrane is a cell-free solution with high concentrations of cell-derived cytokines and growth factors. When combined with hyaluronic acid hydrogel it showed, in a murine wound model, accelerated wound closure through re-epithelialization and decreased wound contraction [90]. From the reported studies, the wide field of applications of HA in skin healing processes appears clearly evident. HA and its derivatives have been tested either alone or combined with numerous other molecules (see Table 1) in order to promote wound healing by influencing proliferative, remodeling and angiogenetic processes.

### 4.4. Vascular Tissue

HA is an important component of vascular tissue. HA has proved active in promoting the migration and subsequent activation of tissue macrophages and neutrophils which, once activated, are able to secrete angiogenic and mitogenic factors essential to the formation of the granulation tissue which is the basis of the angiogenesis [92].

Given its role in angiogenesis, HA has recently been adopted by vascular tissue engineering for several different applications. In many studies, this material was proposed as a promoting factor for vascular graft endothelialization and for vascular substitute. Considering the above-mentioned data, HA was investigated for its role in inducing complete vascular regeneration directly in vivo by the formation of the vascular conduit. In particular, Hyaff-11^®^ biomaterial was utilized to obtain tubules of 2 mm in diameter (50 μm thick) to be used as a vascular prosthesis in animal experimental models [93,94] A segment of rat aorta (2 cm) was incised and the Hyaff tubule, 2 mm in diameter and 2 cm in length, was anastomized, first proximally, then distally in an end-to-end fashion [95]. Similar experiments were performed on the pig carotid artery and on the rat vena cava [96,97]. Regeneration of neo-vascular tissue originated from proximal and distal anastomotic sites, growing inside the tube without signs of infiltration into the prosthesis wall and converging in the middle. All these studies demonstrated the ability of HA-based prostheses to be a useful temporary scaffold for guided artery and vein regeneration in rat and pig models [93,94].

Other studies on vascular grafts focused on HA haemocompatibility and tested its performance when combined with other absorbable and permanent material. A multilayer polyelectrolyte film based on chitosan and HA seeded with mesenchymal stem cells, showed good biocompatibility and induced a fibroblastic morphology in these cells, making this material interesting for the production of vascular substitutes [98]. Moreover, an in vitro study demonstrated that nanofibrous structures made of HA oligosaccharides promoted endothelial cell (EC) proliferation, whereas high molecular weight HA inhibited proliferation. The scaffolds had no detectable degree of haemolysis and coagulation, suggesting the possibility of its use as engineered vascular tissue scaffolds [99]. The luminal surface of expanded polytetrafluoroethylene grafts treated with HA has an improved haemocompatibility without mechanical property changes and without significant cytotoxic effects. This HA layer reduces blood clotting and platelet activation, therefore, this type of product is a promising candidate material for cardiovascular grafts [100]. A HA micro-strip patterned titanium (Ti) surface was used to co-culture vascular smooth muscle cells and endothelial cells. In this in vitro study, a better EC coverage, functional factor release and anti-shedding functions were proved [101].

Another application in vascular tissue engineering for HA may be represented by the promotion of vascularized tissue and microvessels’ formation for tissue ischemia therapy, tissue replacement or even for drug factory models. The hydrogels have ideal mechanical properties for fabricating vascularized dense tissues in vitro. Low molecular weight HA derivatives within gelatin-based hydrogel have proven to promote endothelial cell motility [102]. Another study on an ischemia mouse model demonstrated the therapeutic effects of these fabricated vessel constructions. Specifically, co-aligned human umbilical vein endothelial cells and human adipose stem cells arranged in a biodegradable catechol-conjugated HA hydrogel exhibited enhanced cell to cell contacts, gene expression and secretion of angiogenic and anti-inflammatory paracrine factors [103].

Finally, HA can possibly be used for cell-based therapies. An in vivo and in vitro study investigating the role of HA in embryonic stem cell differentiation toward a smooth muscle cell lineage has proven that remodeling the HA microenvironment is a critical step in directing stem cell differentiation toward a vascular lineage, suggesting a potential role of HA for treatment of vascular diseases [104]. In this section, we have attempted to outline that HA has been proposed for cell-based therapies, to create vascularized tissue and stimulate microvessels’ formation for tissue ischemia therapy, but also as a promoting factor for vascular graft endothelialization and as a vascular substitute. In fact, we believe that these reconstructed vascular tissues are of potential interest to the reconstructive surgery for clinical use and look forward to further development of the use of HA in this field.

### 4.5. Peripheral Nerve

HA has found widespread use in peripheral nerve tissue engineering, supporting nerve outgrowth, differentiation, and proliferation on different substrates. HA hydrogels demonstrated the ability to enhance the survival rates and proliferation of neural precursors, showing great potential for peripheral nerve regeneration approaches [105,106,107] and therapeutic potentials for the central nervous system [108,109,110]. HA hydrogels have biological and mechanical properties able to induce differentiation and proliferation of neural progenitors, opening a new path for therapies targeting neurodegenerative diseases [111,112]. HA can be mixed with natural biopolymers, mainly collagen thanks to the similar nature of the two biomaterials. In fact, Zhang et al. cultured neural stem cells embedded in HA/collagen conduits to promote the regeneration of a 5 mm facial nerve gap in rabbits [113]. HA and chitosan were used successfully is another combination in peripheral nerve regeneration. Li et al., treated peripheral nerve crush injury in a rat model using chitosan conduits combined with HA [114], and Xu et al. used an injectable chitosan/HA biodegradable hydrogel for the regeneration of peripheral nerve injury [115]. Other combinations of HA and biodegradable synthetic polymers were described, such as PLGA and poly-L-lysine, showing a promising potential to control the delivery of drugs for axonal regrowth after spinal cord injury in vitro [116] and in vivo [117]. The possibility to decrease the inflammatory response activated by electroconductive polymers taking advantage of high biocompatibility of HA was also studied in the field of neural tissue engineering. Wang et al. developed a novel porous conductive scaffold, incorporating conductive hyaluronic acid (HA) doped-poly(3,4-ethylenedioxythiophene) (PEDOT-HA) nanoparticles into a chitosan/gelatin (Cs/Gel) matrix, and demonstrated the ability of this engineered construct to support cell adhesion, survival, proliferation, and synapse growth for the application in nerve tissue regeneration [118]. Young JL and Schmidt CE electrochemically coated electrodes with biocompatible and non-cell adhesive hyaluronic acid (HA) to reduce cellular adhesion for potential use in neural prostheses, and demonstrated the possibility to minimized adhesion and migration of fibroblasts and astrocytes to the prostheses [119].

From all the cited studies (Table 1) it appears that HA can play a role also in the therapy of peripheral nerve injury when used in the form of hydrogel or in association either with natural molecules (collagen, chitosan) or with synthetic polymers (PLGA, Ply-L-Lysine) thus confirming an additional unexpected capacity of interacting with specific growth factors involved in peripheral nerve regeneration.

### 4.6. Adipose Tissue

The clinical need for the reconstruction of soft tissue defects due to deep burns, surgical resection, or trauma has stimulated the research in the field of adipose tissue engineering by seeding preadipocytes or adipocyte stem precursors in HA-based scaffolds that could be implanted in animal models for their capacity to generate new fat. The use of HA as a bioresorbable scaffold for adipose tissue engineering has been investigated by many authors [120]. Its clinical use was mainly devoted to the correction of soft-tissue defects in plastic and reconstructive surgery practice. Adipose cells were cultured onto HA materials and implanted in vivo [121]. Tan, et al. developed an injectable thermo-responsive HA gel. They injected HA compounds in the subcutaneous layer of athymic mice and showed in situ gel formation up to 5 days [122].

The main problem was the rapid reabsorption of HA after in vivo implantation, because of its biological properties [123,124]. HA-based (Hyaff-11^®^) pre-adipocyte seeded scaffold was grafted in subcutaneous pockets of patients showing graft survival for up to 16 weeks [125]. The final results of this study showed that the proposed composite scaffolds did not permit adipose tissue formation with deficient angiogenic infiltration [126]. The research was then shifted to increasing the adipogenic properties of HA gels adding drugs or cytokines.

Fan et al. proposed to stimulate adipose tissue growing into HA hydrogels through aqueous Diels–Alder chemistry. The HA hydrogel was functionalized to obtain a gradual release of dexamethasone over a two-week period [127]. Their in vitro results showed an increase of Adipose Stem Cells in groups functionalized with dexamethasone compared HA gels after 14 days. Magnetic HA nano-spheres able to deliver and release dexamethasone in response to magnetic stimuli were also developed [128] and in vitro studies clearly demonstrated increased viability in cell cultures subjected to magnetic impulses. Following these studies, the controlled release of drugs or bioactive molecules continued to be investigated in regenerative medicine. The possibility of combining different bioactive biomaterials was another important field of investigation in adipose tissue engineering. A three-dimensional scaffold able to stimulate adipose tissue development was created crosslinking collagen type I (derived from bovine Achilles) and HA (derived from bovine vitreous humor). Collagen-HA scaffold increased gene expression of adipsin, an enzyme involved in lipid metabolism predominately in mature adipocytes [129]. The biological explanation of these results could be associated with the ability of HA to aid adipogenesis by hastening cell-contacted growth arrest prior to adipogenic conversion [130].

The combination of collagen and elastin for adipose tissue engineering was also investigated by many authors. Preadipocytes seeded on collagen-coated with elastin scaffolds demonstrated the ability to enhance cell proliferation, infiltration, and adhesion [131]. Adipose Stem Cells were seeded onto gelatin-HA cryogel scaffolds and the engineered constructs were implanted into the subcutaneous pocket of two separate animal models: murine and porcine. Acellular cryogels were also implanted for comparison. The relative gene expression of adipocyte-specific genes (PPAR-g, LPL, aP2, and leptin) was investigated, showing that they were significantly greater in the seeded gelatin-HA scaffolds than acellular scaffolds at weeks 2, 4, and 8 in both animal models. Another very interesting new finding was the ability of Gelatin-HA cryogel (with or without cells) to exhibit positive CD-31 staining at eight weeks and adequate porosity for vascularization in the acellular scaffold implants [132]. To summarize, HA was tested in many studies for adipose tissue engineering alone or combined with other bioactive material or drugs or cytokines (Table 1), in order to avoid rapid resorption or even better to promote adipose tissue replacement.

### 4.7. Cancer Therapy

The roles of HA, HAS, Hyal, and HA receptors in cancer biology are complex and mediated by HA receptors expressed in cancer cells [133]. Hence, it was suggested that HA was proposed as a drug carrier or to design nanoparticles or lposomes for its biocompatibility, biodegradability and on the basis of CD44 ability to internalize HA [134,135,136,137]. Carrier drug systems are considered promising cancer therapeutics for the delivery of cytotoxic drugs and many in vivo studies have proven the safety and efficacy of targeted therapy with HA-anti cancer drugs [138,139]. Moreover, HA and its derivatives are promising materials for liposomes functionalization which can delay the release of drugs and enhance local bioavailability. Additionally, HA has recently been proposed to detect CD44 in the diagnosis of specific tumors and recently targeted molecular imaging with HA as specific magnetic resonance contrast agents have been suggested for the diagnosis and treatment of CD44-overexpressing cancer [140,141]. Up to now, cancer therapy with HA-anti-tumoural conjugates appears to be a potentially successful approach in the near future if technical difficulties are resolved. However, a better understanding of the role of HAS, Hyal in cancer biology may lead to their successful clinical usage for cancer treatment. In fact, it has been shown that in many types of solid tumors, increased synthesis of HA by cancer cells or by tumor stromal cells is correlated with tumor growth and metastasis [9,142]. Recent studies have confirmed that the over-expression of HAS2 promotes tumor progression in breast, ovarian, bladder, colorectal, pancreatic and lung carcinoma and resistance to chemotherapy [143,144,145,146,147,148]. On the other hand, it was proved that inhibiting HA synthesis inhibits also metastasis of carcinoma cells in some types of tumors [144,149]. However, other studies reported that HAS3 that was associated with tumor progression, downregulate in early tumor development. For these reasons, it was proposed as a prospective prognostic biomarker and a novel therapeutic target in urothelial carcinoma of the upper urinary tract and urinary bladder [150]. In other types of cancers, progression is not influenced by the HA accumulation but rather by its fragmentation [151]. The degradation of HA in a broad range of its molecular sizes is stimulated by Hyals and tissue ROS that are abundant in tumor microenvironments. In particular, the over-expression of Hyal-1 and Hyal-2 was reported during cancer metastasis in many in vitro and in vivo studies [152,153] and recently it was suggested that HA fragments promote cancer progression via Hippo-Yap signaling [154]. However, the role of Hayl-3 in cancer progression is controversial since some studies have demonstrated its prevention of tumor growth [142], whereas others reported an increased amount of the molecule in some solid tumors [155]. Based on emerging evidence, it was also suggested that specific size ranges of low MW HA exert differential effects on tumor cell survival and growth as recently reviewed by Tavianatou AG et al. [17]. MW HA binds to ECM molecules and cell receptors activating signaling networks for angiogenesis, cell proliferation and extravasation in the microenvironment surrounding metastatic lesions [156]. Small HA fragments are associated with more aggressive solid tumors and recent studies have proven that antibodies or small HA oligomers that inhibit their binding to HA cell receptors are effective in disrupting invasion of tumor cells [157,158,159]. In fact, as previously reported, HA of various MW interact with its two major cell surface receptors, CD44 and RHAMM that act independently or as co-receptors to trigger downstream signaling which enhances tumor progression [160]. However, adaptations in the HA receptor genes CD44 and RHAMM were found in resistant tumors, in breast and colon carcinomas [31]. The correlation between CD44 and tumourigenicity is not absolute since discordant results have been reported [161,162]. In particular, several isoforms of CD44 (CD44v) are up-regulated mostly by cancer cells and necessary for cell cancer invasion. In particular, several isoforms of CD44 (CD44v) are up-regulated mostly by cancer cells and necessary for cell cancer invasion [163]. Thus these receptors have been identified as targets in the treatment of specific cancers directly associated with CD44 since the inhibition of the interaction HA-receptors might result in complete abrogation of tumor progression [164]. Only a few of the described therapeutics have been tested clinically and no HA-based drug delivery systems for human anticancer therapies are actually in clinical use. However, they clearly demonstrated the potential for future use as cancer therapy.

## 5. Conclusions

In this review, we reported the main aspects of the biological roles of HA and the several clinical applications of this polysaccharide which for decades was considered to be only a structural component of the extracellular matrix of skin, joints, eye and many other tissues. With the increasing interest of many biologists, in the last two decades, the view of the role of HA has changed dramatically. The discovery of cell receptors of HA is of fundamental importance in understanding its biological role in certain cell types where it is able to stimulate a cascade of events such as cell motility, adhesion and proliferation. The biosynthesis of this polysaccharide is also unique. Unlike other glycosaminoglycans that are synthesized within the cell, and as reported in the review, HA is synthesized by three transmembrane enzymes at the inner side of the plasma membrane and then extruded directly into the extracellular space. The biological effects of HA sometimes appear contradictory and the discrepancy can be explained by the diversity of the HA preparations used for biomedical applications. However, many aspects of HA metabolism and of its mechanism of action need to be investigated to improve the great number of its applications.

The first clinical use of HA occurred in the 1950s in the field of ophthalmic surgery but nowadays the most widespread use is in arthritis, where it replaces the pathological synovial fluids. The expanding clinical use of HA has stimulated the interest of industry which has provided the preparation of numerous derivatives in order to increase the residence time in the joint cavity. Interestingly with minor chemical modifications of the molecule it has been possible to create highly biocompatible polymers, such as the benzyl ester (Hyaff^®^ biomaterials) that can be used to manufacture non-woven meshes, gauzes, membranes and tubes. Cells seeded into these biomaterials give rise to several tissue substitutes such as dermis, cartilage and bone. Recently, small tubes of Hyaff-11^®^ have been used in rat and pig experimental models as temporary guides for the in vivo reconstruction of the vascular wall. These research studies have shown encouraging results, which open future perspectives for biomedical applications of HA-derivatives. However, these new generations of biocompatible and bio-reabsorbable polymers should be further developed to improve their in-situ performances.

## Figures and Tables

**Table 1:** Medical and tissue engineering hyaluronic acid (HA) applications.

Tissue	Application	Principal Products/Devices	References
*Cartilage*	Osteoarthritis	Synvisc ^®^, injectable solution, (Hyalan G-F20, Sanofi, USA)	[44]
	Osteoarthritis	Hyalgan^®^, injectable solution (Fidia, Italy)	[45,46,47]
	Osteoarthritis	Supartz^®^, injectable solution (Seikagaku, Japan)	[48]
	Osteoarthritis	Orthovisc^®^ and Monovisc^®^, injectable solutions (Anika, USA)	[49,50]
	Osteoarthritis	Durolane^®^, injectable solution, (Smith and Nephew, USA)	[51]
	Osteoarthritis	HYADD^®^ (Fidia, Italy), injectable solution	[52]
	Osteoarthritis	HA-Chitlac^®^, injectable solution	[53,54,55]
	Cartilage regeneration	Hyaff11^®^ (Fidia, Italy), non-woven meshes	[56,57]
*Ocular tissues*	Surgical aid in ophthalmic interventions	Healon^®^, solution (Abbott, USA)	[59,60]
	Tear supplement	Systane^®^, (Alcon, USA)	[63,64,65]
	Tear supplement	Optive Fusion™, (Allergan, USA)	[63,64,66]
*Skin*	Skin substitutes	Hyaff11^®^-non woven meshes (FIDIA, Italy;)	[78,79]
	Skin Substitute	HA-chondroitin sulphate, nanofibrous scaffold	[88]
		HA methacrylated gel	[89]
	Wound healing devices	Cross-linked chitosan-HA-based hydrogels	[91]
		HA-corn-starch-propolis film dressing	[82]
		HA-Fe^+^-based, hydrogel	[83]
		HA-sodium alginate hydrogel	[84]
		HA-polycaprolactone/EGF, nanofibrous scaffold	[87]
		HA solubilized amnion membrane	[90]
*Vascular tissue*	Vascular substitute	Hyaff11^®^ tubes (FIDIA, Italy)	[93,94]
		HA-chitosan biofilm	[92]
		HA-gelatin hydrogel	[102]
		HA-PTFE hydrogel	[100]
		HA-titanium	[101]
		Catechol-conjugated HA hydrogel	[103]
*Adipose tissue*	Tissue substitute	Hyaff-11^®^ non-woven meshes (Fidia, Italy)	[125,126]
		HA-dexamethasone hydrogel	[127,128]
		HA-collagen hydrogel	[129,130]
		HA-collagen-elastin-based gelatin	[131]
*Peripheral Nerve*	Assisted Regeneration devices	HA/collagen composed conduits	[113]
		HA/chitosan composed conduits	[114]
		HA/chitosan injectable hydrogel	[115]
		HA/PLGA/poly-L-lysine based hydrogel	[116,117]
		PEDOT-HA-gelatin matrix	[118]
